# A Prospective Study on Serum Methylmalonic Acid and Homocysteine in Pregnant Women

**DOI:** 10.3390/nu8120797

**Published:** 2016-12-08

**Authors:** Rihwa Choi, Sunkyu Choi, Yaeji Lim, Yoon Young Cho, Hye Jeong Kim, Sun Wook Kim, Jae Hoon Chung, Soo-young Oh, Soo-Youn Lee

**Affiliations:** 1 Department of Laboratory Medicine and Genetics, Samsung Medical Center, Sungkyunkwan University School of Medicine, 81 Irwon-ro, Gangnam-gu, Seoul 06351, Korea; rihwa.choi@samsung.com; 2 Biostatistics and Clinical Epidemiology Center, Samsung Medical Center, 81 Irwon-ro, Gangnam-gu, Seoul 06351, Korea; sunkyu.choi@sbri.co.kr; 3 Biostatistics Team, Samsung Biomedical Research Institute, 81 Irwon-ro, Gangnam-gu, Seoul 06351, Korea; yaeji.lim@pknu.ac.kr; 4 Division of Endocrinology and Metabolism, Department of Medicine, Thyroid Center, Samsung Medical Center, Sungkyunkwan University School of Medicine, 81 Irwon-ro, Gangnam-gu, Seoul 06351, Korea; yoonung2@hanmail.net (Y.Y.C.); eyedr53@schmc.ac.kr (H.J.K.); sunwooksmc.kim@samsung.com (S.W.K.); jaeh.chung@samsung.com (J.H.C.); 5 Department of Obstetrics and Gynecology, Samsung Medical Center, Sungkyunkwan University School of Medicine, 81 Irwon-ro, Gangnam-gu, Seoul 06351, Korea; ohsymd.oh@samsung.com

**Keywords:** methylmalonic acid, homocysteine, vitamin B12, pregnancy

## Abstract

This study aimed to investigate serum methylmalonic acid (MMA) and homocysteine levels and to assess their effects on pregnancy and neonatal outcomes. Serum MMA and homocysteine levels in 278 pregnant Korean women, determined by liquid chromatography–tandem mass spectrometry in each trimester, were compared with those of previous studies in other ethnic groups. We investigated the association between MMA and homocysteine status with pregnancy and neonatal events: gestational diabetes, preeclampsia, gestational age at delivery, preterm birth, small for gestational age, neonatal birth weight, and congenital abnormalities. The median (range) MMA level was 0.142 (0.063–0.446) µmol/L and homocysteine level was 10.6 (4.4–38.0) µmol/L in pregnant women. MMA levels were significantly higher in the third trimester than during other trimesters (*p* < 0.05), while homocysteine levels were not. No significant association was observed between MMA or homocysteine levels and any of the maternal or neonatal outcomes examined. Future studies are needed to assess the associations among maternal serum concentrations of MMA and homocysteine, and maternal and neonatal outcomes.

## 1. Introduction

Vitamin B12, a water-soluble micronutrient which is essential for hematologic and neurologic processes, serves as a cofactor in the remethylation of homocysteine to methionine and in the conversion of l-methylmalonyl-CoA to succinyl-CoA [1]. Vitamin B12 deficiency is an important nutritional problem worldwide as subclinical deficiency affects well-defined risk groups [2]. The recognition and treatment of vitamin B12 deficiency is critical since it is a reversible cause of bone marrow failure and demyelinating nervous system disease in the general population [3]. For pregnant women, maternal serum vitamin B12 concentration has been reported to gradually decline throughout normal pregnancy with the lowest concentration reached in late gestation, and maternal vitamin B12 deficiency has been associated with an increased risk of adverse pregnancy outcomes (e.g., neural tube defects, preterm delivery, and intrauterine growth retardation) indicating the importance of sufficient vitamin B12 intake/status during pregnancy for optimal fetal development and growth [4,5,6,7].

In order to reliably diagnose vitamin B12 deficiency, a combination of several markers associated with vitamin B12 metabolism could be used in place of a single vitamin B12 measurement [2]. Because of the limitations of assays that directly measure vitamin B12 such as poor standardization between different laboratories, different methods or platforms, low sensitivity and specificity, etc., measurement of methylmalonic acid (MMA), homocysteine, or both is used to confirm vitamin B12 deficiency in untreated patients. An elevated level of MMA is more sensitive and specific for diagnosis, since homocysteine level also increases in clinical folate deficiency [3]. The levels of both MMA and total homocysteine are markedly elevated in the vast majority (>98%) of patients with clinical B12 deficiency including those who have only neurologic manifestations of deficiency (i.e., who are not anemic) [3].

Various techniques are used for MMA and homocysteine analyses; these include high performance liquid chromatography (HPLC), gas chromatography–mass spectrometry (GC-MS), liquid chromatography–mass spectrometry (LC-MS), and LC–tandem mass spectrometry (LC-MS/MS) for MMA and enzyme-linked immunosorbent assay, fluorescence polarization immunoassay, chemiluminescent immunometric assay, enzyme-linked immunosorbent assay, radioimmunoassay, HPLC, GC-MS, LC-MS, and LC-MS/MS for homocysteine [2]. Different analytical detection methods have different sensitivities and specificities which could affect the measurement of MMA and homocysteine [2].

Although some researchers have worked to identify the association between maternal MMA and homocysteine levels with pregnancy and neonatal outcomes in different populations [1,8,9,10,11], no reliable data have been collected on a large study population for either MMA or homocysteine levels in pregnant women in East Asian populations including Koreans. Only reports about homocysteine levels in pregnant women in East Asian populations have so far been published [11,12,13].

Therefore, in this prospective study, we measured serum MMA and homocysteine levels simultaneously in pregnant women in Korea using LC-MS/MS. Furthermore, we assessed MMA and homocysteine levels in each trimester and took into account various maternal demographic characteristics. We also investigated the association between maternal serum MMA and homocysteine levels and negative pregnancy and neonatal outcomes.

## 2. Materials and Methods

### 2.1. Study Population

The target population of this study was comprised of pregnant women living in South Korea throughout their pregnancy who visited in our institution from April 2012 to September 2013. During the study period, we recruited 282 pregnant women. Inclusion criteria were as follows: pregnant women, aged 21–50 years at study entry. Exclusion criterion was twin pregnancy by ultrasound at study entry. From 282 pregnant women, we excluded 4 women who had twin pregnancies. Thus, we ultimately enrolled a total of 278 women and their babies.

This study was conducted according to the guidelines laid down in the Declaration of Helsinki, and all procedures involving human subjects were approved by the Institutional Review Board of our institute (SMC 2011-12-041-001). The subjects provided written consent for their participation in the study.

### 2.2. Data Collection

Information about sociodemographic characteristics, smoking, alcohol consumption during pregnancy and during the 4 weeks prior to the last menstrual period, concurrent medical diseases, medications prescribed by doctors, and obstetrical and gynecological histories including parity and type of pregnancy (spontaneous versus artificial) were gathered through general questionnaires at the first visit for prenatal consultation and also from electronic medical records. Multivitamin or folate supplementation was also investigated. For all the women included in the study, the pre-pregnancy body mass index (BMI) was obtained from the self-reported weight and height recorded during the first prenatal consultation. The first trimester BMI was used as a proxy for pre-pregnancy BMI if the pre-pregnancy body weight was unknown. Gestational age was determined according to the last menstrual period or based on the ultrasonographic findings in the first trimester. Pregnancy and neonatal outcomes were obtained from hospital medical records.

### 2.3. Pregnancy and Neonatal Outcomes

Small for gestational age (SGA) babies were defined as those with birth weights below the 10th percentile for their gestational age as determined by birth weight percentile nomograms (national data from Korean Health Insurance Review and Assessment Service 2009). Gestational diabetes (GDM) was defined according to the Carpenter and Coustan criteria [14]. Preeclampsia was defined as the new onset of hypertension (≥140/90 mm·Hg on two separate occasions ≥4 h apart) and proteinuria (≥300 mg/24 h) [15]. Congenital anomaly was defined when a major or minor structural anomaly of the baby was identified prenatally or at birth.

### 2.4. Laboratory Analyses

Blood samples were collected from subjects in red stoppered plain tubes (Becton Dickinson Co., Franklin Lakes, NJ, USA) in the fasting state. Approximately 250 µL of serum was separated and immediately stored at −20 °C until the moment of analysis. Serum MMA and homocysteine levels were analyzed using HPLC (Waters Corporation, Milford) and an API-4000 MS/MS mass spectrometer (Applied Biosystems). Both intra- and inter-assay imprecision were <10% of the coefficient of variation. The accuracy of the homocysteine assay was assured by the Proficiency Testing/Quality Management Program of the Unites States College of American Pathologists. Serum vitamin B12 (cyanocobalamin) levels were measured by electrochemiluminescence immunoassay using the Roche E-170 procedure (Roche, Mannheim, Germany).

### 2.5. Statistical Analysis

Categorical variables are presented as frequencies and percentages. The chi-squared test was used to compare categorical variables. Because age, pre-pregnancy BMI, serum MMA and homocysteine levels, gestational age at delivery and birth weight were not normally distributed, we used nonparametric methods. To assess the association between demographic factors and MMA and homocysteine levels, we used correlation analysis for continuous variables, *t*-tests, or analysis of variance for categorical and continuous variables, and chi-square tests for categorical variables. In the case of a rare event, to assess the association between demographic characteristics and maternal neonatal outcomes, we applied a logistic regression model using Firth’s penalized maximum likelihood estimation method [16]. Variables with a *p* value of less than 0.05 in the univariate analysis were included in the multivariate analysis.

To analyze the association between MMA and homocysteine levels and pregnancy and neonatal outcomes, we applied multiple logistic regressions for dichotomous outcomes and multiple linear regressions for continuous outcomes, adjusting for demographic variables selected from the multivariate analysis of pregnancy and neonatal outcomes and demographic characteristics. Sample size calculation was done with medium Cohen’s effect size (*F*^2^ = 0.15), *n* = 250, with three predictors giving power of 0.9 at 5% level of significance [17]. Statistical analysis was executed using SAS version 9.4 (SAS Institute, Cary, NC, USA). *p* values were corrected by the Bonferroni method in the case of multiple testing and were considered to be significant at the level of 0.05.

## 3. Results

### 3.1. General Characteristics of the Study Population

In total, 278 pregnant Korean women participated in this study. Their median (range) age was 32.0 years (24.0–43.9 years) and median (range) pre-pregnancy BMI was 20.3 kg/m^2^ (15.9–29.6 kg/m^2^). Among them, 93.3% (252/270 women) had more than 12 years of education. The baseline characteristics of the study population are summarized in Table 1. Excluding two pregnant women who miscarried their babies before 20 weeks (10 weeks 0 days and 19 weeks 5 days, respectively), the median (range) gestational age at delivery was 39 weeks 3 days (30 weeks 0 days–41 weeks 3 days) and the median (range) birth weight was 3180 g (1290–4200 g).

Twenty-one (7.6%) pregnant women lacked information regarding pregnancy outcomes due to loss of follow-up.

### 3.2. Serum MMA and Homocysteine Levels in Pregnant Korean Women

**Figure 1:**
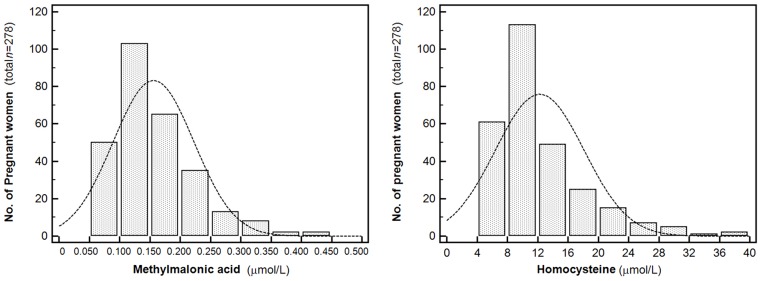
The distribution of methylmalonic acid and homocysteine levels in 278 Korean pregnant women.

The median (range, interquartile range) serum MMA level was 0.142 (0.063–0.446, 0.107–0.187) µmol/L and the median homocysteine was 10.6 (4.4–38.0, 8.2–14.4) µmol/L in pregnant women. Serum MMA and homocysteine levels showed a weak positive correlation (*ρ* = 0.3346, *p* < 0.0001, Supplementary Materials Figure S1). The distribution of MMA and homocysteine levels in study populations were shown in Figure 1.

**Figure 2:**
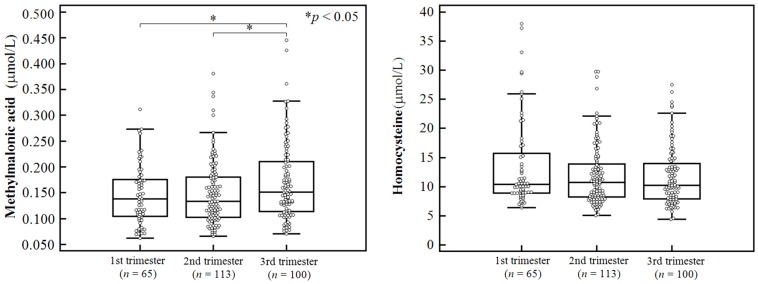
Serum methylmalonic acid and homocysteine levels in all trimesters.

Serum MMA and homocysteine levels in the study population, along with the demographics of the pregnant women, are shown in Table 2. Age and pre-pregnancy BMI were not correlated with either MMA or homocysteine levels (*p* > 0.05). Serum MMA levels were significantly different between the first and third trimester and between the second and third trimester (*p* < 0.05), while homocysteine levels were not significantly different among any of the trimesters. Serum MMA and homocysteine levels in each trimester are shown in Figure 2.

In subgroup analysis for serum MMA, homocysteine, and vitamin B12 levels in 62 pregnant women, median (interquartile range) MMA, homocysteine, and vitamin B12 levels were 0.204 (0.146–0.271) µmol/L, 19.6 (17.2–24.0) µmol/L, and 416.5 (321.0–557.0) pg/mL, respectively. Among 62 pregnant women, only one woman had low serum vitamin B12 concentration with vitamin B12 level 194.0 pg/mL, MMA level 0.344 µmol/L, and homocysteine level 11.3 µmol/L. Results of the subgroup analysis were included as Supplementary Materials Table S2. Cut-off values of 0.400 µmol/L of MMA and 21 µmol/L of homocysteine for vitamin B12 deficiency were applied based on a previous report [3]; the result indicated that none of pregnant women met the criteria for vitamin B12 deficiency, with MMA >0.400 µmol/L and homocysteine >21 µmol/L. Two pregnant women had MMA >0.400 µmol/L, however, their homocysteine levels were <21 µmol/L. Although 26 pregnant women had homocysteine level >21 µmol/L, their MMA levels were ≤0.400 µmol/L, which could also be seen in folate deficiency.

### 3.3. Association between MMA and Homocysteine Levels and Maternal and Neonatal Outcomes

Information about maternal and neonatal outcomes was not available for several pregnant women (Table 1). About 8.3% of women had GDM (23/278), and 2.0% (5/257) experienced and were treated for preeclampsia. Seventeen of 263 babies were preterm (6.5%), 15.2% (39/256) of babies were born small for gestational age, and 6.3% (16/256) of babies were born with congenital anomalies, such as renal anomalies, cardiovascular anomalies, choledochal cysts, periventricular white matter tissue loss, and adrenal cysts. None of babies were born with neural tube defects.

Before assessing the association between serum MMA and homocysteine levels and pregnancy and neonatal outcomes, we examined the association between demographic factors and maternal and neonatal outcomes to identify potential confounding variables, as shown in Supplementary Materials Table S1. At least one demographic factor was associated with each of the maternal and neonatal outcomes, except for multivitamin or folate supplementation. Maternal age was significantly associated with preeclampsia; pre-pregnancy BMI was associated with GDM and preeclampsia; education level was associated with gestational age at delivery, preterm delivery, and baby weight; smoking was associated with preeclampsia; concurrent medical history was associated with gestational age at delivery, preterm delivery, and baby weight; parity was associated with SGA; and type of pregnancy was associated with congenital anomalies (*p* < 0.05). Those variables were included in the multivariate analysis to assess the association between MMA and homocysteine levels and maternal and neonatal outcomes. The association between MMA and homocysteine levels and maternal and neonatal outcomes is summarized in Table 3. No significant association was observed between MMA or homocysteine levels and any of the maternal or neonatal outcomes examined including gestational age at delivery and birth weight of babies (*p* > 0.05).

## 4. Discussion

### 4.1. MMA and Homocysteine Levels in Pregnant Women

In this study, we investigated serum MMA and homocysteine levels in pregnant women along with demographic factors including trimester. Researchers in different countries using different detection methods have endeavored to establish reference levels for MMA and homocysteine in healthy pregnant women while considering the physiological changes that occur during pregnancy (Table 4) [8,9,10,18,19]. The MMA and homocysteine levels in Korean pregnant women were comparable to the findings of previous studies performed in Spain and Ireland, Denmark, The Netherlands, and in women of Nordic descent [8,9,10,18,19]. There was a common trend towards a gradual, significant increase of MMA during pregnancy [8,18,19,20] which is in agreement with the results of this study showing significantly elevated MMA levels in the third trimester. However, our results on homocysteine differed from those of previous studies which reported a gradual, significant increase in levels during pregnancy in a Western population [18,19,20]. Another study performed in pregnant Nepali women reported no significant relationship between homocysteine levels and trimesters, which is in agreement with our results [7]. However, in Nepali women, serum MMA levels were highest in the second trimester, which is not the case in other studies performed in Western populations or in our study [7,8,18,19]. Another study performed in a Japanese population reported that homocysteine levels were only significantly higher in the third trimester compared to in the second trimester [13]. The demand for the vitamin B12 is as high as for folic acid during pregnancy, the B12 stores gradually decrease during pregnancy, hence MMA levels rise [8,21]. The changes in MMA and homocysteine could be due to hemodilution, altered renal function, hormonal changes, changes in the concentration of cobalamin-binding proteins, and materno-fetal cobalamin transfer, which are normal physiological consequences of pregnancy that affect plasma cobalamin concentrations [8,21,22]. Plasma homocysteine levels have been reported to be decreased during normal pregnancy in possible association with the normal increase in the glomerular filtration rate that accompanies pregnancy, the increase in plasma volume and associated hemodilution, and a postulated increased uptake of homocysteine by the fetus [21,22]. As the demand for folic acid increases during pregnancy, without proper supplementation, serum folic acid concentration decreases gradually and its inversely correlated co-marker homocysteine would be expected to increase [11]. In South Korea, folic acid fortification during manufacturing foods itself is not mandatory. However, most physicians recommend folic acid supplementation (400–800 µg/day) in the 3 months before of conception and through the first trimester of pregnancy for every woman who considers pregnancy, as recommended by the U.S. Preventive Services Task Force or the American College of Obstetrics and Gynecology [23]. Since accessibility to health care providers is relatively easy for women in this country, we consider that most pregnant women already take prenatal folic acid supplementation. Although detailed information about intakes of folic acid and vitamin B12 were not available in this study, compliance with the recommendation for the supplementation could explain the result of this study. Socioeconomic factors such as education and job status might be associated with nutrient adequacy [24]. However, no statistical differences were observed between education or job status and serum MMA or homocysteine levels in this study.

### 4.2. MMA and Homocysteine Levels in Maternal and Neonatal Outcomes

In this study, we found no significant association between MMA or homocysteine levels and any of the maternal or neonatal outcomes examined after thoroughly adjusting for potential confounding variables. This could be due to the small number of adverse maternal and neonatal outcomes we observed, which may not have been great enough to provide statistical significance. However, although previous studies have reported that maternal vitamin B12 deficiency is associated with an increased risk of adverse pregnancy outcomes (e.g., neural tube defects, preterm delivery, and intrauterine growth retardation) [1], there have been inconsistent results among studies on MMA and homocysteine levels and maternal and neonatal outcomes [7,9,11,26,27]. Our results concerning homocysteine were comparable with those of a previous study performed in pregnant Korean women which reported no significant association between plasma homocysteine levels and pregnancy outcomes such as preterm delivery, GDM, SGA, placenta abruption or placenta previa, although there was an association between plasma homocysteine levels and preeclampsia [11]. That study found significantly higher plasma homocysteine levels just before delivery as assessed by an automated enzymatic assay in pregnant women with preeclampsia than in normotensive pregnant women [11]. Other studies have reported an association between MMA and homocysteine levels and maternal and neonatal outcomes [9,26,27]. Previous studies were performed with variable specimen types (serum or plasma) and determined the concentrations using GC-MS or LC-MS/MS, which makes direct comparison of their results difficult. A recent review of the cut-off points for a diagnosis of vitamin B12 deficiency in the general population demonstrated the importance of analytical methods; different cut-offs apply when using different analytical methods [2]. The reported cut-off values for MMA in the general population ranged from 0.210 to 0.470 µmol/L [2]. However, the majority of studies were done with GC-MS (77.3%) and they reported very close cut-offs from 0.26 to 0.28 µmol/L [2]. Only one study reported a cut-off value from LC-MS results from 1789 participants in the USA [28]. The reported cut-offs from GC-MS (0.26–0.28 µmol/L) are lower than those from LC-MS (0.35 µmol/L) [28]. In this study, when the cut-off reported from LC-MS in the general population was applied, only four pregnant women showed MMA levels higher than 0.35 µmol/L. When the previously reported cut-off value of 0.400 µmol/L, with 98.0% sensitivity for clinical vitamin B12 deficiency was applied, only two pregnant women were categorized as vitamin B12 deficient. For homocysteine, reported cut-off values range from 10 µmol/L as assessed with HPLC to 21.6 µmol/L as assessed with GC-MS [2]. The review did not include any studies that measured homocysteine levels with LC-MS [2]. When the previously reported cut-off of 21.0 µmol/L with 96.0% of sensitivity for clinical vitamin B12 deficiency was applied, 26 pregnant women had homocysteine level >21.0 µmol/L; however, their MMA levels were <0.400 µmol/L, which could also occur in folate deficiency. None of pregnant women met the criteria for vitamin B12 deficiency with MMA >0.400 µmol/L and homocysteine >21.0 µmol/L. It was difficult to assess the association between vitamin B12 deficiency and maternal and neonatal outcomes due to the small numbers of subjects with vitamin B12 deficiency. Previous studies on the association between MMA and homocysteine levels and maternal and neonatal outcomes used different cut-offs, which could affect the outcomes of the studies [7,12,25]. To elucidate the impact of MMA, homocysteine, and vitamin B23 on maternal and neonatal outcomes, further studies using standardized cut-offs and large numbers of participants are needed.

Because of limited sample volumes, serum vitamin B12 levels could be only performed for some participants and folic acid levels could not be measured. Another limitation of this study is the lack of data on other related laboratory markers such as hematologic markers for anemia. Lack of detailed information about dietary patterns and intakes of folic acid and vitamin B12 such as amount per day, or composition of the multivitamin used is another limitation. Also, results of association between MMA and homocysteine levels and maternal and neonatal outcomes should be considered with caution because the numbers of events were quite small, statistically significant differences were not observed. However, considering that factors that elevated both MMA and homocysteine levels were more sensitive and specific markers than vitamin B12 concentration even in the absence of anemia [3], along with the lack of reliable data on simultaneously assessed MMA and homocysteine levels in pregnant women in East Asian populations, the present study has value in expanding the knowledge base about MMA and homocysteine in pregnant women. The strengths of our study include its prospective design and the use of a sensitive and specific diagnosis method, LC-MS/MS, to simultaneously measure both MMA and homocysteine levels in pregnant women for the first time in the Korean population. We also assessed MMA and homocysteine levels across various maternal demographic characteristics and maternal and neonatal outcomes by multivariate analysis in East Asian pregnant women, to our knowledge, for the first time, although no significant associations were observed.

## 5. Conclusions

In conclusion, in this study, we investigated serum MMA and homocysteine levels simultaneously in pregnant Korean women along with demographic factors for the first time in Korea. There was no independent significant association between MMA or homocysteine levels and any of the maternal or neonatal outcomes examined. Future studies to assess the associations among nutrient supplementation, maternal serum concentration, and maternal and neonatal outcomes may be needed to improve maternal and neonatal health.

## Figures and Tables

**Table 1:** Demographic characteristics and pregnancy outcomes of the sample of 278 Korean pregnant women.

Variables	Participants	
	*n*	%
Trimester at sampling (total)	278	
First	65	(23.4)
Second	113	(40.6)
Third	100	(36.0)
Job (total)	269	
Home maker	90	(33.5)
Any employment	179	(66.5)
Education period (total)	270	
<12 years	18	6.67
≥12 years	252	93.33
Alcohol ingestion (total)	276	
No	273	(98.9)
Yes	3	(1.1)
Smoking (total)	278	
No	277	(99.6)
Yes	1	(0.4)
Concurrent medical history (total)	278	
No	237	(85.3)
Yes	41	(14.7)
Parity (total)	278	
0 (nullipara)	168	(60.4)
≥1	110	(39.6)
Type of pregnancy (total) ^a^	278	
Spontaneous pregnancy	271	(97.5)
Artificial pregnancy	7	(2.5)
Multivitamin or folate supplementation (total)	278	
No	7	(2.5)
Yes	271	(97.5)

Abbreviations: IQR, interquartile range. ^a^ Artificial pregnancies including in vitro fertilization and embryo transfer or controlled ovarian hyperstimulation and intrauterine insemination.

**Table 2:** Association between demographic characteristics and serum methylmalonic acid and homocysteine levels and their status in pregnant Korean women.

Variables	Participants		Methylmalonic Acid Level (µmol/L)			Homocysteine Level (µmol/L)		
	*n*	%	Median	IQR	*p*	Median	IQR	*p*
Trimester at sampling (total)	278				0.03			0.11
First	65	(23.4)	0.138	0.104–0.176		10.4	8.9–15.7	
Second	113	(40.6)	0.133	0.103–0.181		10.7	8.2–13.9	
Third	100	(36.0)	0.151	0.114–0.210		10.3	7.9–14.0	
Job (total)	269				0.47			0.26
Home maker	90	(33.5)	0.144	0.106–0.201		11.3	8.6–15.3	
Any employment	179	(66.5)	0.140	0.107–0.185		10.1	7.9–13.1	
Education period (total)	270				0.93			0.23
<12 years	18	6.67	0.145	0.112–0.195		9.2	7.8–11.3	
≥12 years	252	93.33	0.143	0.107–0.187		10.6	8.2–14.3	
Alcohol ingestion (total)	276				0.34			0.58
No	273	(98.9)	0.142	0.107–0.186		10.6	8.2–14.4	
Yes	3	(1.1)	0.220	–^b, c^		9.0	–^b, c^	
Smoking (total)	278				0.06			0.96
No	277	(99.6)	0.142	0.107–0.186		10.5	8.3–14.4	
Yes	1	(0.4)	0.195	0.155–0.307		11.3	9.0–16.2	
Concurrent medical history (total)	278				0.93			0.37
No	237	(85.3)	0.143	0.108–0.184		10.4	8.2–14.5	
Yes	41	(14.7)	0.134	0.104–0.201		10.6	8.3–14.3	
Parity (total)	278				0.50			0.68
0 (nullipara)	168	(60.4)	0.140	0.107–0.186		10.7	8.5–13.9	
≥1	110	(39.6)	0.144	0.108–0.187		10.3	7.9–14.6	
Type of pregnancy (total) ^a^	278				0.27			0.27
Spontaneous pregnancy	271	(97.5)	0.142	0.107–0.185		10.4	8.2–13.9	
Artificial pregnancy	7	(2.5)	0.189	0.124–0.217		15.1	9.6–18.1	
Multivitamin or folate supplementation (total)	278				0.57			0.41
No	7	(2.5)	0.157	0.096–0.188		14.2	11.1–15.7	
Yes	271	(97.5)	0.142	0.107–0.187		10.4	8.2–13.9	

Abbreviations: IQR, interquartile range. ^a^ Artificial pregnancies including in vitro fertilization and embryo transfer or controlled ovarian hyperstimulation and intrauterine insemination. ^b^ Medians and interquartile ranges were not represented for statistical analysis. ^c^ Interquartile ranges could not be applied because of rare events of the variables. *p*-values for trimester are the result of ANOVA, and *p*-values for other variables are the result of *t*-test.

**Table 3:** Association between serum methylmalonic acid and homocysteine levels and maternal and neonatal outcomes.

	Participants		Methylmalonic Acid Level (µmol/L)				Homocysteine Level (µmol/L)			
Maternal and Neonatal Outcomes	*n*	%	Median	IQR	*p* ^a^	*p* ^b^	Median	IQR	*p* ^a^	*p* ^b^
Gestational diabetes (total)	278				0.40	0.59			0.40	0.47
No	255	(91.7)	0.143	0.109–0.187			10.5	8.3–14.3		
Yes	23	(8.3)	0.134	0.091–0.168			10.6	7.5–14.7		
Preeclampsia (total)	257				0.29	0.30			0.49	0.40
No	252	(98.0)	0.142	0.108–0.186			10.4	8.2–14.1		
Yes	5	(2.0)	0.195	0.151–0.235			11.7	9.9–18.7		
Preterm delivery (total)	263				0.41	0.75			0.27	0.19
No	246	(93.5)	0.142	0.107–0.187			10.4	8.2–13.9		
Yes	17	(6.5)	0.145	0.109–0.184			9.4	7.3–12.6		
Small for gestational age (total)	256				0.12	0.30			0.62	0.64
No	217	(84.8)	0.141	0.108–0.185			10.6	8.2–14.8		
Yes	39	(15.2)	0.161	0.109–0.216			10.2	8.6–13.0		
Congenital abnormality (total)	256				0.69	0.81			0.12	0.15
No	240	(93.8)	0.143	0.107–0.185			10.3	8.2–13.5		
Yes	16	(6.3)	0.150	0.111–0.219			12.7	9.4–18.5		

Abbreviations: IQR, interquartile range. ^a^ *p* value for univariate analysis. ^b^ *p* value for multivariate analysis (demographic variables with a *p* value of less than 0.05 in the univariate analysis were included in the multivariate analysis). Association between serum methylmalonic acid and homocysteine levels and continuous variables, such as gestational age at delivery and birth weight, were not statistically significant and data are not presented.

**Table 4:** Methylmalonic acid and homocysteine levels in pregnant women in previously reported studies.

Ref	Study Region	Study Design	Participants	Specimen	Measurement	Method	Sampling Time	Values	Levels (µmol/L)	Range	Range def.
[8]	Spain and Ireland	Longitudinal	*n* = 92 healthy preg	Plasma	MMA ^a^	GC-MS	Preconception	G- Mean	0.12	(0.09–0.17)	10‰–90‰
							8 wk		0.11	(0.09–0.17)	
							20 wk		0.11	(0.08–0.15)	
							32 wk		0.14	(0.09–0.20)	
							at labor		0.14	(0.09–0.21)	
							Cord blood		0.24	(0.13–0.40)	
[18]	Denmark	Longitudinal	*n* = 406 healthy preg	Plasma	MMA ^a^	GC-MS	18 wk	Median	0.11	(0.06–0.25)	5‰–95‰
							32 wk		0.13	(0.06–0.31)	
							39 wk		0.14	(0.07–0.36)	
							8 wk postpartum		0.16	(0.09–0.30)	
				Plasma	HCY ^a^	GC-MS	18 wk	Median	6.4	(3.6–9.4)	5‰–95‰
							32 wk		7.0	(4.0–9.7)	
							39 wk		7.7	(5.2–12.0)	
							8 wk postpartum		10.8	(6.8–19.3)	
[19]	Denmark	Longitudinal	*n* = 434 healthy preg	Plasma	MMA ^a^	GC-MS	18 wk	Mean	0.11	(0.04–0.29)	Mean ± 1.96 × SD
							32 wk		0.13	(0.05–0.34)	
							39 wk		0.15	(0.06–0.36)	
							8 wk postpartum		0.16	(0.08–0.35)	
				Plasma	HCY ^a^	GC-MS	18 wk	Mean	6.06	(3.34–11.00)	Mean ± 1.96 × SD
							32 wk		6.61	(3.93–11.10)	
							39 wk		7.78	(4.72–12.81)	
							8 wk postpartum		10.99	(5.85–20.64)	
[7]	Nepal	Cross-sectional	*n* = 382 preg	Serum	MMA ^a^	GC-MS	1st trimester	Mean	0.37	(0.32–0.41)	95% CI
							2nd trimester		0.41	(0.36–0.47)	
							3rd trimester		0.39	(0.31–0.48)	
				Serum	HCY	GC-MS	1st trimester	Mean	9.9	(9.1–10.6)	95% CI
							2nd trimester		9.3	(8.6–10.1)	
							3rd trimester		9.4	(8.5–10.3)	
[25]	South India	Cross-sectional	*n* = 360 preg	Plasma	MMA	GC-MS	<14 wk	Median	0.47	(0.28–0.67)	IQR
				Plasma	HCY	GC-MS	<14 wk	Median	9.22	(5.74–15.08)	IQR
[10]	Nordic descent	Longitudinal	*n* = 364 healthy preg	Serum	MMA	GC-MS	17–19 wk	G-Mean	0.10	(0.10–0.11)	95% CI
				Serum	HCY	GC-MS	17–19 wk	G-Mean	4.7	(4.5–4.9)	95% CI
[9]	Netherlands	prospective	*n* = 366 preg (not high-risk preg)	Plasma	MMA	LC-MS/MS	30–34 wk	Median	0.16	(0.13–0.22)	IQR
				Plasma	HCY	LC-MS/MS	30–34 wk	Median	5.5	(4.5–6.7)	IQR
This study	South Korea	Prospective cohort	*n* = 278 preg	Serum	MMA ^a^	LC-MS/MS	5–13 wk	Median	0.13	(0.10–0.18)	IQR
							14–26 wk		0.13	(0.10–0.18)	
							27–40 wk		0.15	(0.11–0.21)	
				Serum	HCY	LC-MS/MS	5–13 wk	Median	10.6	(8.9–15.7)	IQR
							14–26 wk		10.6	(8.2–13.9)	
							27–40 wk		10.2	(7.9–14.0)	

Abbreviations: preg, pregnant women; wk, week of gestation; CI, confidence interval; def. definition; G-mean, Geometric Mean; GC, gas chromatography; HCY, homocysteine; IQR, interquartile range; LC, liquid chromatography; MS, mass spectrometry; MMA, methylmalonic acid; SD, standard deviation. ^a^ These studies have been reported significant differences in methylmalonic acid and/or homocysteine levels at different sampling times.

## Supplementary Materials

The following are available online at http://www.mdpi.com/2072-6643/8/12/797/s1, Figure S1: Correlation between serum methylmalonic acid and homocysteine levels in pregnant Korean women. Serum methylmalonic acid and homocysteine levels showed a weak positive correlation (*ρ* = 0.3346, *p* < 0.0001). The circles denote results of serum methylmalonic acid and homocysteine levels. The filled circle denotes results of a pregnant woman whose serum vitamin B12 < 200 pg/mL, Table S1: Association between basic demographic characteristics and maternal and neonatal outcomes in pregnant Korean women (*p*-values for univariate analysis), Table S2: Vitamin B12 deficiency in 62 pregnant Korean women based on serum methylmalonic acid, homocysteine, and vitamin B12 concentrations using cutoffs reported in literature.

## Abbreviations

The following abbreviations are used in this manuscript:

BMI	body mass index
GC-MS	gas chromatography-mass spectrometry
GDM	gestational diabetes; HPLC, high performance liquid chromatography
LC-MS	liquid chromatography-mass spectrometry
LC-MS/MS	liquid chromatography-tandem mass spectrometry
MMA	methylmalonic acid
SGA	small for gestational age
