# Supplementary material for: A Prospective Study on Serum Methylmalonic Acid and Homocysteine in Pregnant Women
Source: Nutrients. 2016 Dec 8;8(12):797. doi: 10.3390/nu8120797 (PMC5188452; doi:10.3390/nu8120797)
Supplement: Supplementary file 1 [file nutrients-08-00797-s001.docx]

Supplementary Materials: A Prospective Study on Serum Methylmalonic Acid and Homocysteine in Pregnant Women

Rihwa Choi, Sunkyu Choi, Yaeji Lim, Yoon Young Cho, Hye Jeong Kim, Sun Wook Kim, Jae Hoon Chung, Soo-young Oh and Soo-Youn Lee


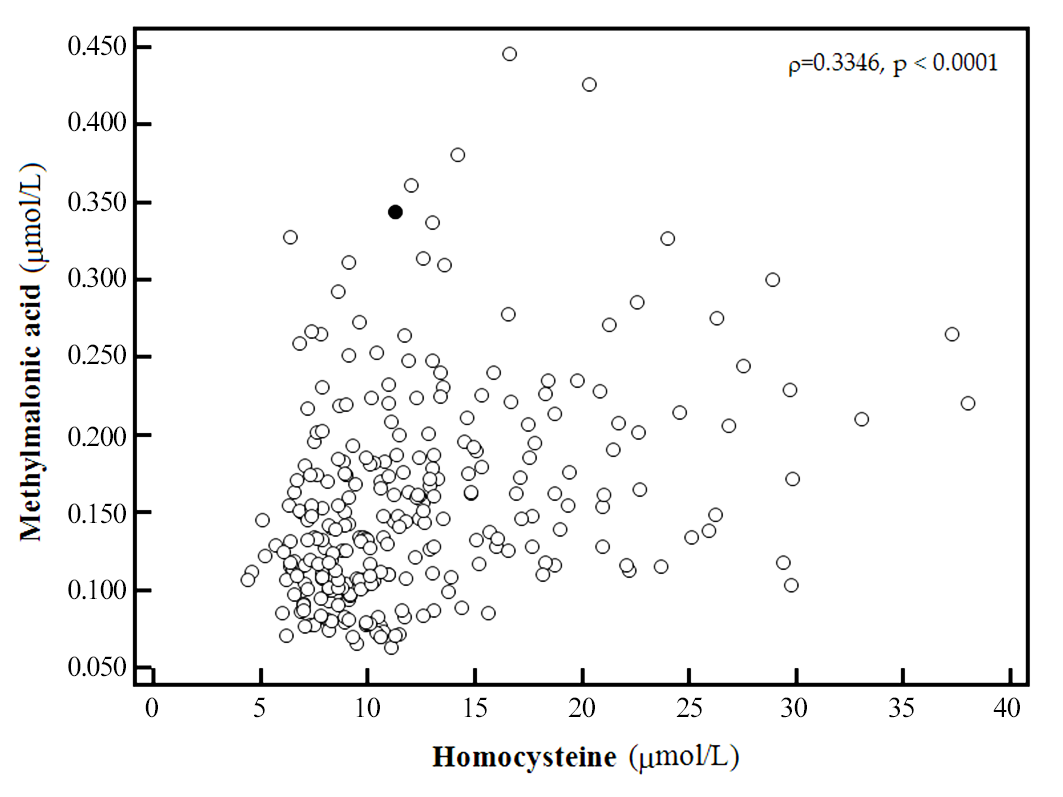


**Figure S1.** Correlation between serum methylmalonic acid and homocysteine levels in pregnant Korean women. Serum methylmalonic acid and homocysteine levels showed a weak positive correlation (*ρ* = 0.3346, *p* < 0.0001). The circles denote results of serum methylmalonic acid and homocysteine levels. The filled circle denotes results of a pregnant woman whose serum vitamin B12 < 200 pg/mL.

**Table S1.** Association between basic demographic characteristics and maternal and neonatal outcomes in pregnant Korean women (*p*-values for univariate analysis).

|  | **GDM** | **Pre-eclampsia** | **Gestational Age at Delivery** | **Preterm Delivery** | **Baby Weight** | **Small for Gestational Age** | **Congenital Anomaly** |
| --- | --- | --- | --- | --- | --- | --- | --- |
| Age | 0.12 | **0.03** | 0.1 | 0.08 | 0.53 | 0.88 | 0.44 |
| Pre-pregnancy BMI | **0.00 ^b^** | **0.00 ^a^** | 0.37 | 0.39 | 0.55 | 0.39 | 0.36 |
| Trimester at sampling | | | | | | | |
| First | Reference | | | | | | |
| Second | 0.17 | 0.14 | **0.00 ^a^** | 0.07 | 0.06 | 0.28 | 0.91 |
| Third | 0.36 | 0.15 | **0.00 ^b^** | **0.02** | 0.13 | **0.02** | 0.4 |
| Job | | | | | | | |
| Home maker | Reference | | | | | | |
| Any employment | 0.29 | 0.53 | 0.84 | 0.76 | 0.75 | 0.09 | 0.06 |
| Education period | | | | | | | |
| <12 years | Reference | | | | | | |
| ≥12 years | 0.64 | 0.26 | **0.00 ^c^** | **0.00 ^c^** | **0.01** | 0.33 | 1 |
| Alcohol ingestion ^d^ | | | | | | | |
| No | Reference | |  |  |  |  |  |
| Yes | 0.99 |  |  |  |  |  |  |
| Smoking | | | | | | | |
| No | Reference | | | | | | |
| Yes | 0.16 | **0.01 ^a^** | 0.18 | 0.11 | 0.4 | 0.99 | 0.99 |
| Concurrent medical history | | | | | | | |
| No | Reference | | | | | | |
| Yes | 0.33 | 0.13 | **0.03** | **0.00 ^c^** | **0.00 ^a^** | 0.86 | 0.22 |
| Parity | | | | | | | |
| 0 (nullipara) | Reference | | | | | | |
| ≥1 | 0.09 | 0.41 | 0.09 | 0.78 | 0.2 | **0.01 ^a^** | 0.32 |
| Type of pregnancy ^e^ | | | | | | | |
| Spontaneous pregnancy | Reference | | | | | | |
| Artificial pregnancy | 0.56 | 0.99 | 0.71 | 0.41 | 0.85 | 0.94 | **0.03** |
| Multivitamin or folate supplementation | | | | | | | |
| No | Reference | | | | | | |
| Yes | 0.56 | 0.99 | 0.72 | 0.99 | 0.87 | 0.59 | 0.99 |

BMI, body mass index; GDM, gestational diabetes. Demographic factors which were significantly associated with maternal and neonatal outcomes (**Bold**) were included in multivariate analysis. ^a^ *p* < 0.01. ^b^ *p* < 0.001. ^c^ *p* < 0.0001. ^d^ Because of rare events (only one pregnant woman ingested alcohol), statistical analyses were performed only for certain outcomes. ^e^ Artificial pregnancies including in vitro fertilization and embryo transfer or controlled ovarian hyperstimulation and intrauterine insemination.

**Table S2.** Vitamin B12 deficiency in 62 pregnant Korean women based on serum methylmalonic acid, homocysteine, and vitamin B12 concentrations using cutoffs reported in literature.

| **Markers** | **Cutoff ^a^** | **Reported Sensitivity ^a^** | **Reported Specificity ^a^** | **Numbers of Vit B12 Deficiency by Trimester** | | | | **Numbers of Subjects** | | |
| --- | --- | --- | --- | --- | --- | --- | --- | --- | --- | --- |
|  |  |  |  | **Total  (*n* = 62)** | **First Trimester  (*n* = 17)** | **Second Trimester  (*n* = 25)** | **Third Trimester  (*n* = 20)** | **MMA > 0.400 µmol/L** | **Hcy > 21 µmol/L** | **Vit B12 < 200 pg/mL** |
| MMA | >0.400 **µ**mol/L | 98% for clinical deficiency | Poor specificity for clinical response in patients with modest elevation of level of MMA (0.300–1.000 **µ**mol/L) | 2 | 0 | 0 | 2 | 2 | **0** | **0** |
| Hcy | >21 µmol/L | 96% for clinical deficiency | Hcy level also increased in clinical folate deficiency and renal insufficiency | 26 | 12 | 7 | 7 | - | 26 | **0** |
| Vit B12 | <200 pg/mL | 65%–95% for proven clinical deficiency; 50% for detecting elevated level of MMA | 50%–60% for clinical response; 80% for detecting elevated level of MMA | 1 | 0 | 1 | 0 | - | - | 1 |

Abbreviations: MMA, methylmalonic acid; Hcy, homocysteine; Vit B12, vitamin B12. ^a^ Data taken from [3].
